# Lipoprotein-associated phospholipase A2 levels, endothelial dysfunction and arterial stiffness in patients with stable coronary artery disease

**DOI:** 10.1186/s12944-021-01438-4

**Published:** 2021-02-14

**Authors:** Konstantinos Mourouzis, Gerasimos Siasos, Evangelos Oikonomou, Marina Zaromitidou, Vicky Tsigkou, Alexis Antonopoulos, Evanthia Bletsa, Panagiota Stampouloglou, Konstantinos Vlasis, Manolis Vavuranakis, Dimitris Tousoulis

**Affiliations:** 1 1st Cardiology Department, Hippokration Hospital, Athens Medical School, National and Kapodistrian University of Athens, Athens, Greece; 2 Medizinische Klinik und Poliklinik I, Klinikum der Universität München, Munich, Germany; 3 Cardiovascular Division, Brigham and Women’s Hospital, Harvard Medical School, Boston, MA USA; 4 Department of Anatomy, Laiko General Hospital, National and Kapodistrian University of Athens, School of Medicine, Athens, Greece

**Keywords:** Lipoprotein-associated phospholipase A2, Endothelial dysfunction, Wave reflections, Coronary artery disease, Arterial stiffness

## Abstract

**Background:**

Lipoprotein-associated Phospholipase A2 (Lp-PLA2), can exert proinflammatory as well as proatherogenic properties on the vascular wall. The current study sought to evaluate the influence of high Lp-PLA2 levels on indices of arterial wall properties in patients with stable coronary artery disease (CAD).

**Methods:**

Three hundred seventy-four consecutive patients with stable CAD (mean age 61 ± 11 years, 89% males) were enrolled in this single-center cross-sectional study. Flow-mediated dilation (FMD) was used to assess endothelial function and augmentation index (AIx) of the central aortic pressure was used to assess reflected waves. ELISA was used to determine Lp-PLA2 serum levels.

**Results:**

After dividing the participants in 3 equal groups based on the tertiles of circulating Lp-PLA2 values, no significant differences were demonstrated between those in the 3rd tertile with Lp-PLA2 values > 138 μg/L, in the 2nd tertile with Lp-PLA2 values between 101 and 138 μg/L and in the 1st tertile (Lp-PLA2 values < 101 μg/L) regarding age, male gender, smoking habits, family history of CAD or history of a previous myocardial infarction, diabetes mellitus, arterial hypertension, hyperlipidemia, duration of CAD and treatment with relevant medication. Importantly, subjects with Lp-PLA2 values in the highest tertile, had significantly reduced FMD values compared to the middle and lower tertile (4.43 ± 2.37% vs. 4.61 ± 1.97% vs. 5.20 ± 2.52% respectively, *P =* 0.03). Patients in the highest tertile of Lp-PLA2 values had significantly higher AIx values (24.65 ± 8.69% vs. 23.33 ± 9.65%, *P =* 0.03), in comparison to the lowest tertile, with Lp-PLA2 values < 101 μg/L. A linear regression analysis showed that Lp-PLA2 values > 138 μg/L negatively correlated to FMD [b = − 0.45 (95% CI: − 0.79 – -0.11), *P =* 0.01] and AIx values [b = 1.81 (95% CI: 0.57–3.05), *P* < 0.001] independently of cofounders like gender, age, diabetes mellitus, arterial hypertension, dyslipidemia, smoking habits, family history of CAD, history of previous myocardial infarction, serum glucose, circulating lipid levels, duration of CAD, antihypertensive medication, antidiabetic drugs, statin therapy and treatment with β-blockers.

**Conclusions:**

Elevated Lp-PLA2 levels relate to endothelial dysfunction and arterial stiffness in patients with stable CAD independently from classical risk factors for CAD, statin use, antihypertensive treatment, and duration of the disease.

**Supplementary Information:**

The online version contains supplementary material available at 10.1186/s12944-021-01438-4.

## Introduction

Coronary artery disease (CAD) is a complex pathology with manifold aspects, which include systematic inflammation, structural changes on the vascular wall, endothelial dysfunction, arterial stiffness and discrete plaque formation within the coronary arteries [1].

Lipoprotein-associated phospholipase A2 (Lp-PLA2), also referred to as platelet-activating factor acetylhydrolase (PAF-AH), is predominantly secreted de novo from inflammatory cells or bound to circulating low density lipoprotein (LDL) and in a much smaller proportion to circulating high density lipoprotein (HDL) [2]. The fact that it is detected in high concentration in atherosclerotic plaques combined with its ability to hydrolyze phospholipids in LDL, which in turn results in proinflammatory and proatherogenic products such as oxidized free fatty acids, have supported the idea that Lp-PLA2 is strongly involved in the acceleration of the atherosclerotic process [3]. The suggestion that the inhibition of Lp-PLA2 could benefit patients with CAD, led to the conduction of randomized, clinical studies such as the Stabilization of Atherosclerotic Plaque by Initiation of Darapladib Therapy (STABILITY) trial [4] and The Stabilization Of pLaques usIng Darapladib-Thrombolysis In Myocardial Infarction 52 Trial (SOLID-TIMI 52) [5]. Although darapladib-induced Lp-PLA2 inhibition could not reduce major adverse cardiovascular events (MACE), a subsequent interleukin (IL)-6 and C-reactive protein (CRP) reduction observed in these studies, may associate with better cardiovascular outcomes [4, 5].

Endothelial dysfunction is well recognized as an early-occuring, fundamental component in the pathophysiology of cardiovascular disease [6]. Endothelial function can be non-invasively estimated by flow-mediated dilation (FMD), typically in the brachial artery. It is an established risk factor which independently predicts cardiovascular events [7]. Impaired vascular wall properties, including wave reflection and arterial stiffness, also possess a prognostic capacity for cardiovascular disease and early appearance of reflected waves in systole associates with reduced coronary perfusion [8, 9].

Since only a few studies have investigated the interplay between non-invasive biomarkers of arterial wall properties and Lp-PLA2 in stable CAD, the current study aims to examine the relationship between Lp-PLA2, endothelial function and arterial stiffness in stable CAD patients.

## Materials and methods

### Study design and population

In this single-center cross-sectional study, 374 consecutive patients (mean age 61 ± 11 years) with history of stable CAD were enrolled, after the application of the exclusion criteria and after signing the consent form. As history of stable CAD was defined the evidence of a narrowing of more than 50% of at least one major epicardial coronary artery in a diagnostic coronary angiography, which was conducted after a positive stress test, angina or chest pain or in the context of an acute coronary syndrome (ACS). Participating subjects were at stable clinical condition after receiving appropriate treatment and/or free of an ACS for the last 6 months before entering the study. The recruitment took place between February 2016 and March 2019. At least two experienced cardiologists interpreted the coronary angiographies. Several demographic and clinical data, among which age, weight, height, blood pressure, the history of diabetes mellitus, arterial hypertension, hyperlipidemia and family history of cardiovascular disease were also recorded. Subjects smoking at least one cigarette/day or those that quitted smoking less than a year from the time of the index coronary angiography were defined as current smokers, patients reporting ever smoking before this point of time were defined as former smokers and those who never consumed tobacco products were defined as non-smokers.

Patients with evidence of reduced left ventricle ejection fraction < 50%, severe valvulopathies, ACS in the last 6 months, chronic kidney failure, osteoporosis, malignant or immunological diseases, under systemic glycorticoid or immunosuppressive therapy were defined as exclusion criteria of the study.

### Coronary angiography

All patients underwent an elective coronary angiography by experienced interventional cardiologists from a radial or femoral arterial access. Vessel stenosis and magnitude of lesions were assessed with a quantitative coronary angiography system. Significant CAD was defined as the narrowing of the luminal diameter of a major epicardial coronary artery ≥50%.

### Assessment of endothelial function

Flow-mediated dilation (FMD) in the brachial artery was used to estimate endothelial dysfunction. The assessment process is previously described [10]. Following a 10 min rest, the examiner scans and measures the right brachial artery in a longitudinal direction, with the use of a linear array ultrasound transducer attached to a Vivid e ultrasound device (General Electric, Milwaukee, (WI), USA). This is followed by an induction of a 5-min reactive hyperemia, by adjusting and inflating a pneumatic cuff distally to the ultrasound transducer to an increased systolic pressure compared to that measured on the forearm. Subsequently to releasing the ischemia cuff, the manual estimation of the diameter of the brachial artery using electronic calipers in intervals of 15 s for 2 min was conducted. The evaluation of FMD was conducted by estimating the % change of the brachial artery diameter of the measurement taken at 60 s after releasing ischemia compared to the measurements taken at baseline (at rest). A consequent measurement was conducted after resting for 10 min, which was used to assess the endothelium-independent dilation (EID), after administrating a single, sublingual dose (400 μg) of glyceryl trinitrate by comparing it with the maximal diameter measured on 2 and 5 min after glyceryl trinitrate administration. All examinations were carried out by the same person; a blinded observer carried out the measurements through the study [10]. A repeatability coefficient of 5% was calculated based on the Bland-Altman method.

### Assessment of wave reflections

Augmentation index (AIx) of the central (aortic) pressure waveform was evaluated to estimate wave reflections. This was achieved by applanation tonometry, which was used to appropriately capture and analyze non-invasively the arterial pulse using a validated acquisition system (SphygmoCor, AtCor Medical, Sydney (NSW), Australia). AIx is an index of the severity of wave reflection and higher values reflect enhanced arterial stiffness. AIx can be strongly influenced from heart rate, which is why a correction of AIx on a stable heart rate of 75 beats per min follows. The calibration of radial pressure waveforms is achieved by sphygmanometrically measuring blood pressure values on the forearm [11].

### Biochemical measurements

After being centrifuged at 906 x g, venous blood samples were stored at − 80 °C until assay. The assay of the circulating levels of Lp-PLA2 was conducted in the biochemistry laboratory of the 1st University Cardiology Clinic of the University of Athens with a commercially available enzyme-linked immunoassay (ELISA) (PLAC test, diaDexus, Inc., San Francisco, (CA), USA). According to the data provided from the manufacturer, the minimum detection limit of the used test is 0.34 μg/L, the intra-essay precision rate lies between 4,3–5,8% of coefficient of variation (%CV) and the inter-assay precision rate ranges from 6.3%CV to 8.7%CV. Commercially enzymatic methods were used to assay circulating lipid and glucose levels.

### Statistical analysis

Normal distribution of the variables was tested with the use of *P-P* plots. Data with normal distribution are expressed as means ± standard deviation and skew variables as median with first and third quartiles. Categorical variables are reported as exact number of participants with the respective proportion in the study (sub)population. An initial analysis in which patients were divided in in groups based on the median of Lp-PLA2 (that being 125 μg/L in the current study) was initially conducted. Differences between these 2 groups were tested with t-test or with Wilcoxon-test for continuous variables with normal and non-normal distribution respectively. Another analysis, in which patients were categorized according to the tertiles of Lp-PLA2 values was conducted (1st tertile: Lp-PLA2 values < 101 μg/L, 2nd tertile: Lp-PLA2 values between 101 and 138 μg/L and 3rd tertile: Lp-PLA2 values > 138 μg/L) Differences between the 3 groups were tested with one way ANOVA and Kruskal-Wallis H Test for continuous variables with normal and non-normal distribution respectively, along with Bonferroni test. Chi-square test was performed to assess differences regarding categorical variables. Linear correlations between continuous variables with non-normal distribution were assessed by Spearman’s correlation coefficient. To furtherly reduce confounding, a multiple linear regression analysis model was used to explore the correlation of Lp-PLA2 levels with AIx and FMD, after adjustment for multiple cofounders. All reported *p*-values correspond to two-sided tests and *P-*values were considered statistically significant at the level of < 0.05. Data were analyzed using SPSS software, version 26.0 (IBM, Chicago, (IL), USA).

## Results

### Baseline characteristics of the study population depending on Lp-PLA2 values

The median value of serum Lp-PLA2 levels in the study population was 125 (96–152) μg/L. Using the median of Lp-PLA2 levels as a threshold, 2 patient groups were formed. Demographic and clinical characteristics between patients who had circulating Lp-PLA2 values < 125 μg/mL and those with ≥125 μg/L are shown in Table S1. A further analysis was consequently conducted with patients being categorized by tertiles of Lp-PLA2 values. The clinical and demographic characteristics and differences between the 3 groups of the study are shown in Table 1.

**Table 1:** Comparison of demographic, clinical and laboratory characteristics based on Lp-PLA2 tertiles

Characteristics	T1 < 101 μg/L	T2 101-138 μg/L	T3 > 138 mg/L	***P*** value
**Male Gender (n, %)**	109 (90)	114 (93)	113 (89)	0.43
**Age (years)**	63 ± 10	62 ± 12	61 ± 11	0.44
**Body mass index (kg/m**^**2**^**)**	28.04 ± .4	28.58 ± 3.79	28.02 ± 3.40	0.43
**Diabetes mellitus (n, %)**	39 (30)	31 (25)	29 (23)	0.28
**Hypertension (n, %)**	96 (78%)	92 (76)	99 (79)	0.88
**Hyperlipidemia (n, %)**	98 (80)	91 (75)	89 (73)	0.25
**Smoking history (n, %)**	100 (82)	102 (84)	102 (84)	0.91
**Current smokers (n, %)**	29 (22)	24 (20)	34 (28)	
**Former smokers (n, %)**	71 (60)	78 (64)	68 (56)	
**Heart Failure (n, %)**	20 (16)	26 (22)	22 (18)	0.57
**Family History for CAD (n, %)**	31 (25)	33 (26)	33 (26)	0.94
**Previous myocardial infarction (n, %)**	52 (43)	47 (39)	58 (46)	0.51
**CAD Duration (months)**	40 (26–55)	47 (32–61)	46 (24–62)	0.15
**Statins (n, %)**	110 (91)	101 (82)	105 (84)	0.19
**β-blockers (n, %)**	83 (68)	80 (66)	93 (74)	0.33
**Antidiabetic agents (n, %)**	32 (27)	30 (25)	26 (21)	0.25
**ACEi or ARBs**	81 (68)	75 (61)	77 (62)	0.51
**Systolic arterial pressure (mm Hg)**	126 ± 20	125 ± 15	128 ± 19	0.42
**Diastolic arterial pressure (mm Hg)**	77 ± 12	77 ± 10	78 ± 10	0.82
**Serum glucose (mg/dL)**	119 ± 44	116 ± 43	110 ± 40	0.30
**Cholesterol (mg/dL)**	156 ± 35^a^	165 ± 40^a^	171 ± 46^a^	0.03
**LDL (mg/dL)**	93 ± 30^a^	100 ± 32^a^	104 ± 35^a^	0.04
**HDL (mg/dL)**	40 ± 10	42 ± 11	41 ± 10	0.57
**Triglycerides (mg/dL)**	129 ± 54	141 ± 74	136 ± 74	0.42
**Lp-PLA2 (μg/L)**	75 (60–92)	120 (113–130)	160 (149–179)	< 0.001
**EID (%)**	14.62 ± 7.51	14.42 ± 5.17	14.05 ± 3.92	0.29
**FMD (%)**	5.20 ± 2.52	4.61 ± 1.97	4.43 ± 2.37	0.03
**AIx (%)**	22.36 ± 8.92^a^	24.33 ± 9.65^a^	24.65 ± 8.69^a^	0.12

Data are presented as mean ± SD or range between q1-q3 or n, (%)

*P* values are calculated using χ2 for categorical independent variables and one way ANOVA or Kruskal-Wallis H test for continuous independent normally distributed and non-normally distributed variables respectively

^a^Bonferroni test showed a significant difference between T3 -T1 at the level of p=0.04

*CAD* Coronary artery disease, *LDL* Low density lipoprotein, *HDL* High density lipoprotein, *Lp-PLA2* Lipoprotein-associated phospholipase A2, *EID* Endothelial-independent dilatation, *FMD* Flow-mediated dilatation, *AIx* Augmentation index, *ACEi* Angiotensin-converting-enzyme, *ARBs* Angiotensin-II -receptor Type 1 blocker

### Comparisons between study groups depending on Lp-PLA2 values

No differences between patients with Lp-PLA2 < 101 μg/L (T1), 101–138 μg/L (T2) and those with Lp-PLA2 values > 138 μg/L (T3) regarding age (63 ± 10 vs.62 ± 12 vs.61 ± 11 respectively, *P =* 0.43), male gender (90% vs. 93% vs. 90% respectively, *P =* 0.44), smoking history (82% vs. 84% vs. 84% respectively, *P =* 0.91), diabetes mellitus (30% vs. 25% vs. 23% respectively, *P =* 0.28) and hyperlipidemia (80% vs. 75% vs. 73% respectively, *P =* 0.25) were observed.

Subjects in T3 had higher total cholesterol levels (171 ± 46 mg/dL vs. 156 ± 35 mg/dL, *P =* 0.03), LDL (104 ± 35 mg/dL vs. 93 ± 30 mg/dL, *P =* 0.04) when compared to the lowest tertile of the study. No significant differences were observed concerning the levels of HDL (*P =* 0.57), triglycerides (*P =* 0.42) or glucose (*P =* 0.30), as well as statin use (*P =* 0.19), treatment with angiotensin converting enzyme inhibitors (ACEi) or angiotensin-II -receptor Type 1 blockers (ARB), (*P =* 0.51) or antidiabetic treatment coverage (*P =* 0.25) in the three groups of the study (Table 1).

**Fig. 1:**
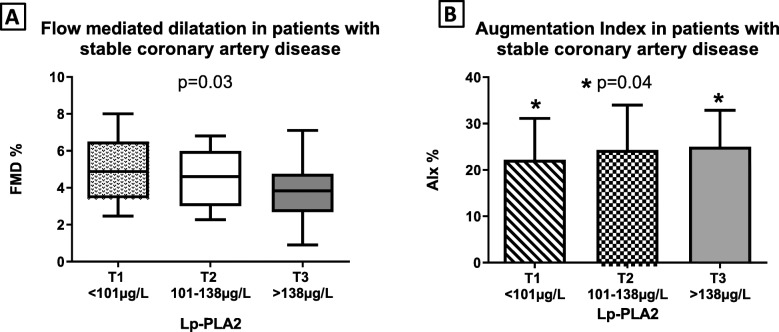
Differences in FMD and AIx values according to Lp-PLA2 levels by tertiles. **a** Box-plots of FMD values in the Lp-PLA2 < 101 μg/L, Lp-PLA2 101–138 μg/L group and Lp-PLA2 > 138 μg/L group in subjects with stable CAD. **b** Bar graph of Aix values in subjects with Lp-PLA2 < 101 μg/L, Lp-PLA2 101–138 μg/L compared to subjects with Lp-PLA2 values > 138 μg/L. AIx differs significantly between 1st and 3rd tertile of Lp-PLA2 values. Lp-PLA2: Lipoprotein-associated phospholipase A2; FMD: flow-mediated dilatation; AIx: Augmentation Index

EID values did not differ between T3, T2 and T1 (*P =* 0.29). More importantly, patients with Lp-PLA2 > 138 μg/L presented with significantly lower FMD (4.43 ± 2.37% vs. 4.61 ± 1.97% vs. 5.20 ± 2.52%, *P =* 0.03) when compared to those with Lp-PLA2 values between 101 and 138 μg/L and those with values < 101 μg/L respectively. AIx values did not differ between the 3 groups (*P = 0.*12). When a separate comparison was conducted, patients in the highest tertile had significantly increased AIx compared to those in the lowest tertile (24.65 ± 8.69% vs. 22.36 ± 8.92 *P =* 0.04) (Fig. 1).

### Factors affecting FMD values

To examine how demographic or clinical factors and biomarkers may affect FMD values, multiple factors were examined in correlation with FMD within the study population (Table S2a). Importantly, a negative linear correlation between FMD and circulating Lp-PLA2 levels (rho = − 0.13, *P =* 0.02) was demonstrated.

Furthermore, the impact of increased Lp-PLA2 on FMD values, independently from other cofounders, was investigated through a multiple linear regression analysis, as shown in Table 2. According to these findings, values of circulating Lp-PLA2 > 138 μg/L correlate to worse FMD values [b = − 0.45 (95% CI: − 0.79 – -0.11), *P =* 0.01] when compared to the lowest tertile, independently of factors such as gender, age, diabetes mellitus, arterial hypertension, dyslipidemia, smoking, family history of CAD or previous myocardial infarction, serum glucose, circulating total cholesterol and LDL levels, duration of CAD, antihypertensive medication, antidiabetic drugs, statin therapy and treatment with β-blockers.

**Table 2:** Multiple linear regression analysis for the association of FMD and AIx with several variables

Variables	Regression analysis for the association of FMD (dependent variable) with Lp-PLA2 after adjustment for multiple classical risk factors for CAD			Regression analysis for the association of AIx (dependent variable) with Lp-PLA2 after adjustment for multiple classical risk factors for CAD		
	b coefficient	95% CI	*P* value	b coefficient	95% CI	*p*-value
**Gender**	− 0.30	− 1.48 – 0.87	0.61	−7.16	− 11.30 – 3.03	< 0.001
**Age (years)**	− 0.01	− 0.04 – 0.03	0.69	0.22	0.11 – − 0.34	< 0.001
**Duration of CAD (months)**	0.01	− 0.01 – 0.02	0.59	0.01	−0.05 – 0.07	0.68
**Arterial hypertension**	0.76	−0.18 – 1.69	0.11	−0.94	−4.12 – 2.25	0.56
**Diabetes mellitus**	−0.25	−2.07 – 1.57	0.79	5.89	0.01–11.78	0.05
**Hyperlipidemia**	−0.20	−1.10 – 0.70	0.66	1.43	−1.74 – 4.59	0.37
**Smoking history**	−0.18	−0.64 - 0.28	0.44	1.66	0.07–3.25	0.04
**Family history for CAD**	0.74	−0.07 – 1.55	0.07	−1.26	−4.12 – 1.60	0.39
**Previous MI**	0.08	−0.60 – 0.77	0.81	0.07	−2.40 – 2.53	0.96
**Statins**	−1.12	−2.32 – 0.08	0.07	−1.98	−5.96 – 1.99	0.33
**Antidiabetic agents**	−0.01	−1.80 – 1.78	0.99	−5.49	−11.31 – 0.33	0.06
**ACEi/ARBs**	−0.18	−0.94 – 0.57	0.63	1.61	−1.13 – 4.35	0.25
**β-blockers**	0.04	−0.72 – 0.81	0.91	0.29	−2.46 – 3.04	0.83
**Cholesterol (mg/dL)**	0.01	−0.001 – 0.02	0.55	0.01	−0.04 – 0.06	0.66
**LDL (mg/dL)**	−0.01	−0.03 – 0.01	0.19	0.02	−0.04 – 0.08	0.50
**Glucose (mg/dL)**	0.01	−0.01 – 0.01	0.73	−0.01	−0.04 – 0,02	0.54
**Lp-PLA2 > 138 μg/L**	− 0.45	−0.79 - -0.11	0.01	1.81	0.57 - 3.05	< 0.001

For categorical variables, reference category was set the absence of male gender, diabetes mellitus, hyperlipidemia, smoking history, arterial hypertension, family history for CAD, previous myocardial infarction, statin therapy, treatment with ACEi/ARBs, antidiabetic agents or with β-blockers and Lp-PLA2 < 101 μg/L (1st tertile). *AIx* Augmentation Index, *FMD* Flow-mediated dilatation, *CAD* Coronary artery disease, *Lp-PLA2* Lipoprotein-associated phospholipase A2, *ACEi* Angiotensin converting enzyme inhibitors, *ARBs* Angiotensin II receptor blockers, *MI* Myocardial infarction

### Factors affecting AIx values

To examine how demographic or clinical factors and biomarkers may affect AIx values, multiple factors were examined in association with AIx within the study population (Table S2b). AIx positively correlated with age (*r* = 0.32, *p* < 0.001), total cholesterol (*r* = 0.21, *P =* 0.01), LDL (*r* = 0.14, *P =* 0.02), and HDL levels (*r* = 0.13, *P =* 0.02). More importantly, AIx values correlated to circulating Lp-PLA2 levels (rho = 0.11, *P =* 0.04).

The impact of increased Lp-PLA2 on AIx values, independently from other cofounders, was furtherly investigated in a multiple linear regression analysis model as demonstrated in Table 2. According to the analysis, factors like age, gender, diabetes mellitus and smoking correlated positively with AIx values, independently from the other classical risk factors for CAD and circulating lipids like total cholesterol, LDL and glucose levels. Moreover, the highest tertile, that being Lp-PLA2 values > 138 μg/L, was associated with impaired AIx values [b = 1.81 (0.57–3.05), *P* < 0.001] when compared to patients with Lp-PLA2 values < 101 μg/L.

## Discussion

Wave reflection and arterial stiffness are broadly used as indices of vascular wall dysfunction and can be used as clinical biomarkers and predictors in the context of cardiovascular disease, along with other pro-inflammatory regulators [12]. In the current study, worse AIx values were associated with elevated circulating Lp-PLA2 levels and this observation was consistently demonstrated, independently of other confounders, among which lipid lowering and antihypertensive agents and the duration of CAD. Only a limited series of studies exists in the literature which have investigated the relationship between circulating Lp-PLA2 and arterial stiffness indices. Schnabel et al. demonstrated that higher AIx measurements were related to increased circulating proinflammatory mediators, including Lp-PLA2 in 2409 participants of the Framingham Heart Study [13]. Further studies showed that Lp-PLA2 values > 234.5 μg/L correlate to augmented arterial stiffness and could hold a prognostic role in patients with stable CAD [14]. The hydrolysis of oxidized-LDL by Lp-PLA2 may be the joining link between Lp-PLA2 unfavorable effects on wave reflection and arterial stiffness [15]. Acting as a proinflammatory and proatherogenic agent, Lp-PLA2 suggestively affects not only the markers of vascular function of the peripheral but also the coronary arterial tree, in terms of wave reflection and abnormal coronary flow reserve respectively [16].

In this study, it was also shown that patients with stable CAD, presenting with higher circulating Lp-PLA2 levels, have also impaired endothelial function, as assessed by FMD in the brachial artery. Only a few studies exist in the literature which examine the relation between Lp-PLA2 and endothelial dysfunction in the context of stable CAD, despite the fact that Lp-PLA2 is considered to act as a mediator between systemic inflammatory state, severity of CAD and plaque progression and vulnerability [17]. Yang EH et al. have previously demonstrated that patients with subclinical or mild CAD, with serum Lp-PLA2 concentrations above the second tertile, presented with significantly worse intracoronary function, in terms of impaired flow and vasoconstriction [18]. Moreover, another study showed that increased intracoronary Lp-PLA2 secretion correlated strongly with intracoronary dysfunction and the atheromatic volume of coronary plaques [19]. Supportive evidence deriving from smaller patient cohorts strengthen the suggestion of an Lp-PLA2 and endothelial function interplay, which correlates to the global coronary atherosclerotic burden assessed by coronary flow reserve [15]. Lp-PLA2 is considered as an important regulator between systemic inflammation and the progression of atherosclerosis, something which is also reflected on endothelial dysfunction surrogate markers like increased adhesion molecules and impaired NO-secretion [17].

Lp-PLA2 is part of a complex interplay between systemic inflammation, functional and structural changes of the arterial wall changes, which in whole could furtherly accelerate the progression of CAD. Early studies have suggested that Lp-PLA2 could be used as a useful tool for better risk stratification in CAD [20]. Several guidelines suggest cut-off values to identify patients in greater risk for cardiovascular events. For example Lp-PLA2 values ≥200 μg/L in combination with other biomarkers, e.g. CRP may predict a higher atherosclerotic cardiovascular disease risk [21]. Darapladib is a pharmaceutical agent that can attenuate plasma Lp-PLA2 activity [22]. Findings from 2 large randomized, clinical studies were not conclusive on the additional benefit after Lp-PLA2 inhibition with darapladib on patients after ACS [5] or with stable CAD regarding the midterm prognosis for MACE or in delaying the progression of vulnerable plaque formation [4]. This could be partly attributed to the fact that most of the patients participating in STABILITY and SOLID-TIMI 52 trials were concurrently receiving statins, which in high doses exert a capacity of lowering Lp-PLA2 up to 20%, something that also adds to their known anti-inflammatory properties [23]. However, a further analysis of these data suggests that Lp-PLA2 pharmaceutic suppression associates with a concomitant IL-6 and CRP reduction, which in turn may beneficially influence cardiovascular outcomes by reducing total or major coronary events [24]. Based on these mixed findings, many international guidelines do not currently strongly support measurements of Lp-PLA2 to better characterize the absolute risk for MACE in patients with CAD. However, Lp-PLA2 may, synergistically to CRP, contribute to better risk assessment and guided management in patients with specific characteristics and complementary to established risk factors, e.g. patients with very high baseline LDL-values, with diabetes mellitus or history of cardiovascular events [25]. To add to that, other studies show that elevated CRP and Lp-PLA2 levels correlate to increased circulating von Willebrand factor (vFW) [26]. vVF is an important regulator of systemic inflammation and thrombosis and its interplay with other inflammatory mediators like Lp-PLA2 may be of great significance in the progression of CAD [27, 28]. Recent animal studies also show a favorable effect of darapladib on the preservation of retina-barrier, indicating novel possible treatment options for high risk populations (e.g. patients with diabetes mellitus) [29]. Specially designed studies which will assess the supplementary value of Lp-PLA2 in risk stratification or its inhibition as an adjuvant therapeutic option specifically in these subgroups were not yet conducted. Further clinical studies should follow to evaluate the additional benefit in risk assessment in specific CAD patient subgroups, e.g. patients with diabetes mellitus, high cholesterol or LDL-levels despite statin treatment or recurrent ischemic events by assessing Lp-PLA2 conjointly to CRP, other surrogate markers of systemic inflammation and impaired vascular wall function, including FMD and AIx.

## Study strengths and limitations

One of the strengths of this single-center, cross-sectional study is that endothelial function was assessed with brachial FMD, a biomarker which appears more sensitive on estimating endothelial dysfunction in CAD in comparison to other methods. Another advantage is that a statistically significant predictive role for Lp-PLA2 regarding FMD and AIx values was demonstrated after adjusting for numerous clinically relevant confounders in the multiple linear regression model. More importantly, all classical cardiovascular risk factors were adjusted in this model. These results highlight the association of Lp-PLA2 with other surrogate markers of inflammation and arterial wall properties in patients with stable CAD and its role in the pathophysiology of CAD.

The study had however limitations. Despite adjustment for numerous confounders, the risk of bias and residual confounding may have not been avoided. Existing guidelines suggest that Lp-PLA2 levels ≥200 ng/ml should be used to reclassify patients as high-risk, who need a more aggressive lipid lowering approach. Only 14 participants had Lp-PLA2 values ≥200 μg/L in the study population. A reason for this observation could be that the great majority of patients were already on statin therapy and had not extremely high LDL-levels (mean value 92 ± 32 mg/dL). In comparison with previous studies, an “all-comer” population of stable CAD-patients and not only those with high LDL values was included in the study, which may have added to this observation. Differences regarding AIx values between hypertensive and non-hypertensive patients or patients with smoking history and non-smokers were not significant, as shown in other studies. This could be attributed to the fact that our study focused on the relationship between Lp-PLA2, FMD and AIx and not on exploring differences of AIx between subgroups of patients with CAD. Other confounders, including differences in medical treatment, duration of CAD or comorbidities may have contributed to this finding.

## Conclusion

Elevated Lp-PLA2 levels relate to endothelial dysfunction and arterial stiffness in patients with stable CAD independently from classical risk factors for CAD, statin use, antihypertensive treatment and duration of the disease.

## Supplementary Information

**Additional file 1: Figure S1.** Differences in FMD and AIx values according to Lp-PLA2 levels. Panel A: Box-plots of FMD values according to Lp-PLA2 levels. Panel B: Box-plots of AIx values according to Lp-PLA2 levels. Lp-PLA2: lipoprotein-associated phospholipase A2; FMD: Flow-mediated dilatation; AIx: augmentation index.

**Additional file 2: Table S1.** Comparison of demographic, clinical and laboratory characteristics between patients with Lp-PLA2 ≥ 125 μg/L versus patients with Lp-PLA2 < 125 μg/L.

**Additional file 3: Table S2.** a Comparison of demographic, clinical and laboratory characteristics between patients regarding FMD values. b Comparison of demographic, clinical and laboratory characteristics between patients regarding AIx values.

**Additional file 4: Table S3.** Multiple linear regression analysis for the association of FMD and AIx with several variables.

## Data Availability

The datasets used and/or analysed during the current study are available from the corresponding author on reasonable request.
