# Supplementary material for: Lipoprotein-associated phospholipase A2 levels, endothelial dysfunction and arterial stiffness in patients with stable coronary artery disease
Source: Lipids Health Dis. 2021 Feb 14;20:12. doi: 10.1186/s12944-021-01438-4 (PMC7883455; doi:10.1186/s12944-021-01438-4)
Supplement: Supplementary file 1 — Additional file 1: Figure S1. Differences in FMD and AIx values according to Lp-PLA2 levels. Panel A: Box-plots of FMD values according to Lp-PLA2 levels. Panel B: Box-plots of AIx values according to Lp-PLA2 levels. Lp-PLA2: lipoprotein-associated phospholipase A2; FMD: Flow-mediated dilatation; AIx: augmentation index. [file 12944_2021_1438_MOESM1_ESM.docx]

| **Supplementary Table 1:** Comparison of demographic, clinical and laboratory characteristics between patients with Lp-PLA2 ≥ 125 μg/L versus patients with Lp-PLA2 < 125 μg/L | | | | | |
| --- | --- | --- | --- | --- | --- |
| **Characteristics** | | **Patients with Lp-PLA2 < 125 μg/L**  **(n=187)** | **Patients with Lp-PLA2 ≥ 125 μg/L**  **(n=187)** | ***P-*value** |  |
| **Male Gender (n, %)** | | 168 (90) | 168 (90) | 0.91 |  |
| **Age (years)** | | 62 ± 10 | 60 ± 10 | 0.20 |  |
| **Body mass index (kg/m^2^)** | | 28.02 ± 3.69 | 28.21 ± 3.51 | 0.31 |  |
| **Diabetes mellitus (n, %)** | | 56 (30) | 43 (23) | 0.14 |  |
| **Hypertension (n, %)** | | 143 (77) | 144 (78) | 0.84 |  |
| **Hyperlipidemia (n, %)** | | 141 (77) | 136 (73) | 0.34 |  |
| **Smoking history (n, %)** | | 150 (82) | 154 (84) | 0.53 |  |
|  | **Current smoker (n, %)** | 40 (21) | 47 (25) |  |  |
|  | **Former smoker (n, %)** | 110 (61) | 107 (59) |  |  |
| **Heart Failure (n, %)** | | 34 (19) | 34 (19) | 0.96 |  |
| **Family History for CAD (n, %)** | | 47 (25) | 50 (27) | 0.70 |  |
| **Previous myocardial infarction (n, %)** | | 77 (41) | 80 (43) | 0.76 |  |
| **Duration of CAD (months)** | | 43.5 (28-57) | 46.5 (27-63) | 0.34 |  |
| **Statins (n, %)** | | 161 (87) | 155 (84) | 0.41 |  |
| **β-blockers (n, %)** | | 119 (64) | 137 (74) | 0.10 |  |
| **Antidiabetic agents (n, %)** | | 50 (27) | 38 (20) | 0.13 |  |
| **ACEi or ARBs** | | 116 (63) | 117 (64) | 0.85 |  |
| **Systolic arterial pressure (mm Hg)** | | 124 ± 14 | 128 ± 20 | 0.09 |  |
| **Diastolic arterial pressure (mm Hg)** | | 76 ± 11 | 77 ± 9.45 | 0.77 |  |
| **Serum glucose (mg/dL)** | | 118 ± 46 | 105 ± 34 | 0.02 |  |
| **Cholesterol (mg/dL)** | | 157 ± 40 | 169 ± 41 | 0.02 |  |
| **LDL (mg/dL)** | | 94 ± 30 | 101 ± 31 | 0.07 |  |
| **HDL (mg/dL)** | | 41 ± 10 | 40 ± 11 | 0.79 |  |
| **Triglycerides (mg/dL)** | | 134 ± 55 | 136 ± 76 | 0.72 |  |
| **Lp-PLA2 (μg/L)** | | 99 (73-115) | 154 (140-172) | <0.001 |  |
| **EID (%)** | | 14.56 ± 6.47 | 14.10 ± 4.78 | 0.52 |  |
| **FMD (%)** | | 4.89 ± 2.07 | 4.44 ± 2.19 | 0.04 |  |
| **AIx (%)** | | 23.06 ± 9.47 | 25.21 ± 8.70 | 0.03 |  |
| Data are presented as mean ± SD or range between q1-q3 or n (%)  CAD: coronary artery disease; LDL: low density lipoprotein; HDL: high density lipoprotein; Lp-PLA2: lipoprotein-associated phospholipase A2; EID: Endothelial-independent dilatation; FMD: Flow-mediated dilatation; AIx: augmentation index; ACEi: angiotensin converting enzyme inhibitors, ARBs: Angiotensin II receptor blockers | | | | | |
