# Supplementary material for: Lipoprotein-associated phospholipase A2 levels, endothelial dysfunction and arterial stiffness in patients with stable coronary artery disease
Source: Lipids Health Dis. 2021 Feb 14;20:12. doi: 10.1186/s12944-021-01438-4 (PMC7883455; doi:10.1186/s12944-021-01438-4)
Supplement: Supplementary file 2 — Additional file 2: Table S1. Comparison of demographic, clinical and laboratory characteristics between patients with Lp-PLA2 ≥ 125 μg/L versus patients with Lp-PLA2 < 125 μg/L. [file 12944_2021_1438_MOESM2_ESM.docx]

| **Supplementary Table 2a:** Comparison of demographic, clinical and laboratory characteristics between patients regarding FMD values | | | | | |
| --- | --- | --- | --- | --- | --- |
| **Characteristics** | | **Yes** | **No** | ***P-*value** |  |
| **Male Gender** | | 4.67 ± 2.28% | 5.37 ± 2.57%, | 0.33 |  |
| **Diabetes mellitus** | | 4.48 ± 2.01% | 4.78 ± 2.33%, | 0.27 |  |
| **Hypertension** | | 4.75 ± 2.28% | 4.51 ± 2.14%, | 0.89 |  |
| **Hyperlipidemia** | | 4.69 ± 2.20% | 4.76 ± 2.42 | 0.33 |  |
| **Smoking history** | | 4.61 ± 2.12% | 5.15 ± 2.18% | 0.11 |  |
|  | **Current smokers*** | 4.80 ± 2.32% | 5.15 ± 2.81% | 0.45 |  |
|  | **Former smokers*** | 4.55 ± 2.04 % | 5.15 ± 2.81% | 0.08 |  |
| **Heart Failure** | | 4.61 ± 2.01 | 4.67 ± 2.30 | 0.87 |  |
| **Family History for CAD** | | 5.22 ± 2.54% | . 4.52 ± 2.11 | 0.08 |  |
| **Previous myocardial infarction** | | 4.72 ± 2.32 | 4.79 ± 2.33 | 0.77 |  |
| **Statins** | | 4.63 ± 2.19 | 5.22 ± 2.63 | 0.12 |  |
| **β-blockers** | | 4.63 ± 2.27 | 4.82 ± 2.15 | 0.49 |  |
| **Antidiabetic agents** | | 4.59 ± 2.01 | 4.74 ± 2.33 | 0.62 |  |
| **ACEi or ARBs** | | 4.71 ± 2.32 | 4.69 ± 2.12 | 0.96 |  |
| **Supplementary Table 2b:** Comparison of demographic, clinical and laboratory characteristics between patients regarding AIx values | | | | |  |
| **Male Gender** | | 23.29 ± 9.15% | 29.34 ± 6.99% | <0.001 |  |
| **Diabetes mellitus** | | 23.36 ± 8.16% | 24.05 ± 9.49% | 0.53 |  |
| **Hypertension** | | 24.03 ± 9.15% | 23.26 ± 9.17% | 0.53 |  |
| **Hyperlipidemia** | | 23.80 ± 9.35% | 24.06 ± 8.50 % | 0.82 |  |
| **Smoking history** | | 24.03 ± 9 % | 22.89 ± 8.90 % | 0.38 |  |
|  | **Current smokers*** | 24.05 ± 9.35% | 22.89 ± 8.90 % | 0.46 |  |
|  | **Former smokers*** | 24.02 ± 8.89 | 22.89 ± 8.90 % | 0.39 |  |
| **Heart Failure** | | 23.49 ± 8.70 | 25.03 ± 9.67 | 0.26 |  |
| **Family History for CAD** | | 22.56 ± 10.01% | 24.31 ± 8.75% | 0.13 |  |
| **Previous myocardial infarction** | | 23.28 ± 8.92 | 24.26 ± 9.35 | 0.33 |  |
| **Statins** | | 23.43 ± 9.82 | 25.66 ± 8.33 | 0.11 |  |
| **β-blockers** | | 24.70 ± 8.96 | 23.35 ± 8.96 | 0.21 |  |
| **Antidiabetic agents** | | 22.99 ± 8.56 | 24.04 ± 9.35 | 0.36 |  |
| **ACEi or ARBs** | | 23.84 ± 9.03 | 23.67 ± 9.38 | 0.87 |  |
| Data are presented as mean ± SD or range between q1-q3  CAD: coronary artery disease; Lp-PLA2: lipoprotein-associated phospholipase A2; FMD: Flow-mediated dilatation; AIx: augmentation index; ACEi: Angiotensin converting enzyme inhibitors; ARBs: angiotensin-II -receptor Type 1 blocker  * compared to non-smokers | | | | | |
