# Supplementary material for: Lipoprotein-associated phospholipase A2 levels, endothelial dysfunction and arterial stiffness in patients with stable coronary artery disease
Source: Lipids Health Dis. 2021 Feb 14;20:12. doi: 10.1186/s12944-021-01438-4 (PMC7883455; doi:10.1186/s12944-021-01438-4)
Supplement: Supplementary file 3 — Additional file 3: Table S2. a Comparison of demographic, clinical and laboratory characteristics between patients regarding FMD values. b Comparison of demographic, clinical and laboratory characteristics between patients regarding AIx values. [file 12944_2021_1438_MOESM3_ESM.docx]

| **SupplementaryTable 3** Multiple linear regression analysis for the association of FMD and AIx with several variables | | | | | | |
| --- | --- | --- | --- | --- | --- | --- |
| **Variables** | **Regression analysis for the association of FMD (dependent variable) with Lp-PLA2 after adjustment for multiple classical risk factors for CAD** | | | **Regression analysis for the association of AIx (dependent variable) with Lp-PLA2 after adjustment for multiple classical risk factors for CAD** | | |
|  | b coefficient | 95% CI | p-value | b coefficient | 95% CI | p-value |
| **Gender** | -0.36 | -1.28 - 0.56 | 0.44 | -5.73 | -9.29 - -2.17 | 0.001 |
| **Age (years)** | -0.020 | -0.05 - 0.01 | 0.12 | 0.24 | 0.14 - 0.33 | 0.001 |
| **Duration of CAD (months)** | -0.004 | -0.02 - 0.01 | 0.58 | 0.009 | -0.04 - 0.06 | 0.72 |
| **Arterial hypertension** | 0.47 | -0.22 - 0.17 | 0.18 | 0.48 | -2.12 - 3.07 | 0.72 |
| **Diabetes mellitus** | -0.61 | -1.91 - 0.68 | 0.35 | 1.39 | -3.39 - 6.18 | 0.57 |
| **Hyperlipidemia** | 0.03 | -0.67 - 0.74 | 0.92 | 1.78 | -0.87 - 4.42 | 0.19 |
| **Smoking history** | -0.18 | -0.54 - 0.17 | 0.31 | 1.85 | 0.52 - 3.18 | 0.01 |
| **Family history for CAD** | 0.56 | -0.04 - 1.17 | 0.07 | -0.29 | -2.60 - 2.01 | 0.80 |
| **Previous MI** | 0.04 | -0.50 - 0.58 | 0.89 | -0.58 | -2.62 - 1.46 | 0.58 |
| **Statins** | -1.23 | -2.12 - -0.33 | 0.07 | -1.16 | -4.44 - 2.13 | 0.49 |
| **Antidiabetic agents** | 0.45 | -0.82 - 1.71 | 0.49 | -1.45 | -6.13 - 3.22 | 0.54 |
| **ACEi/ARBs** | -0.24 | -0.81 - 0.33 | 0.40 | 1.07 | -1.10 - 3.25 | 0.33 |
| **β-blockers** | -0.025 | -0.61 - 0.56 | 0.93 | -0.42 | -2.68 - 1.84 | 0.71 |
| **Cholesterol (mg/dL)** | 0.01 | -0.01 - 0.01 | 0.95 | 0.01 | -0.03 - 0.05 | 0.53 |
| **LDL (mg/dL)** | -0.01 | -0.02 - 0.003 | 0.12 | 0.02 | -0.03 - 0.07 | 0.36 |
| **Glucose (mg/dL)** | 0.003 | -0.005 - 0.01 | 0.48 | -0.02 | -0.05 - 0.01 | 0.16 |
| **Lp-PLA2 ≥ 125 μg/L** | -0.55 | -1.07 - -0.03 | 0.04 | 2.07 | 0.06 - 4.07 | 0.04 |
| For categorical variables, reference category was set the absence of male gender, diabetes mellitus, hyperlipidemia, smoking history, arterial hypertension, family history for CAD, previous myocardial infarction, statin therapy, treatment with ACEi/ARBs, antidiabetic agents or with β-blockers and Lp-PLA2 < 125 μg/L. AIx: Augmentation Index; FMD: Flow-mediated dilatation; CAD: coronary artery disease; Lp-PLA2: Lipoprotein-associated phospholipase A2; ACEi: Angiotensin converting enzyme inhibitors; ARBs: Angiotensin II receptor blockers; MI: myocardial infarction | | | | | | |
