# Supplementary material for: Lipoprotein-associated phospholipase A2 levels, endothelial dysfunction and arterial stiffness in patients with stable coronary artery disease
Source: Lipids Health Dis. 2021 Feb 14;20:12. doi: 10.1186/s12944-021-01438-4 (PMC7883455; doi:10.1186/s12944-021-01438-4)
Supplement: Supplementary file 4 — Additional file 4: Table S3. Multiple linear regression analysis for the association of FMD and AIx with several variables. [file 12944_2021_1438_MOESM4_ESM.pptx]

## Slide 1
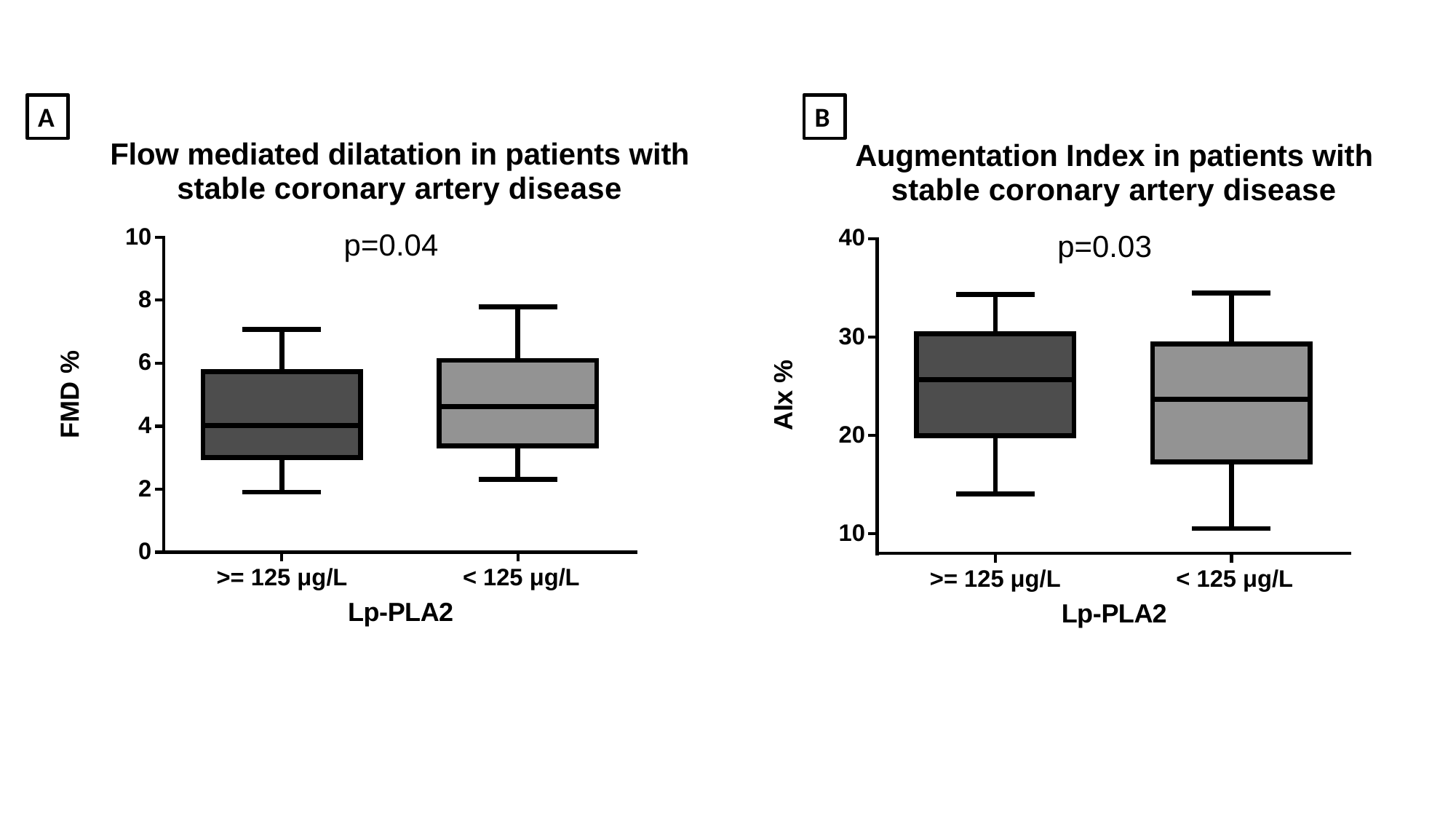

A
B

## Slide 2
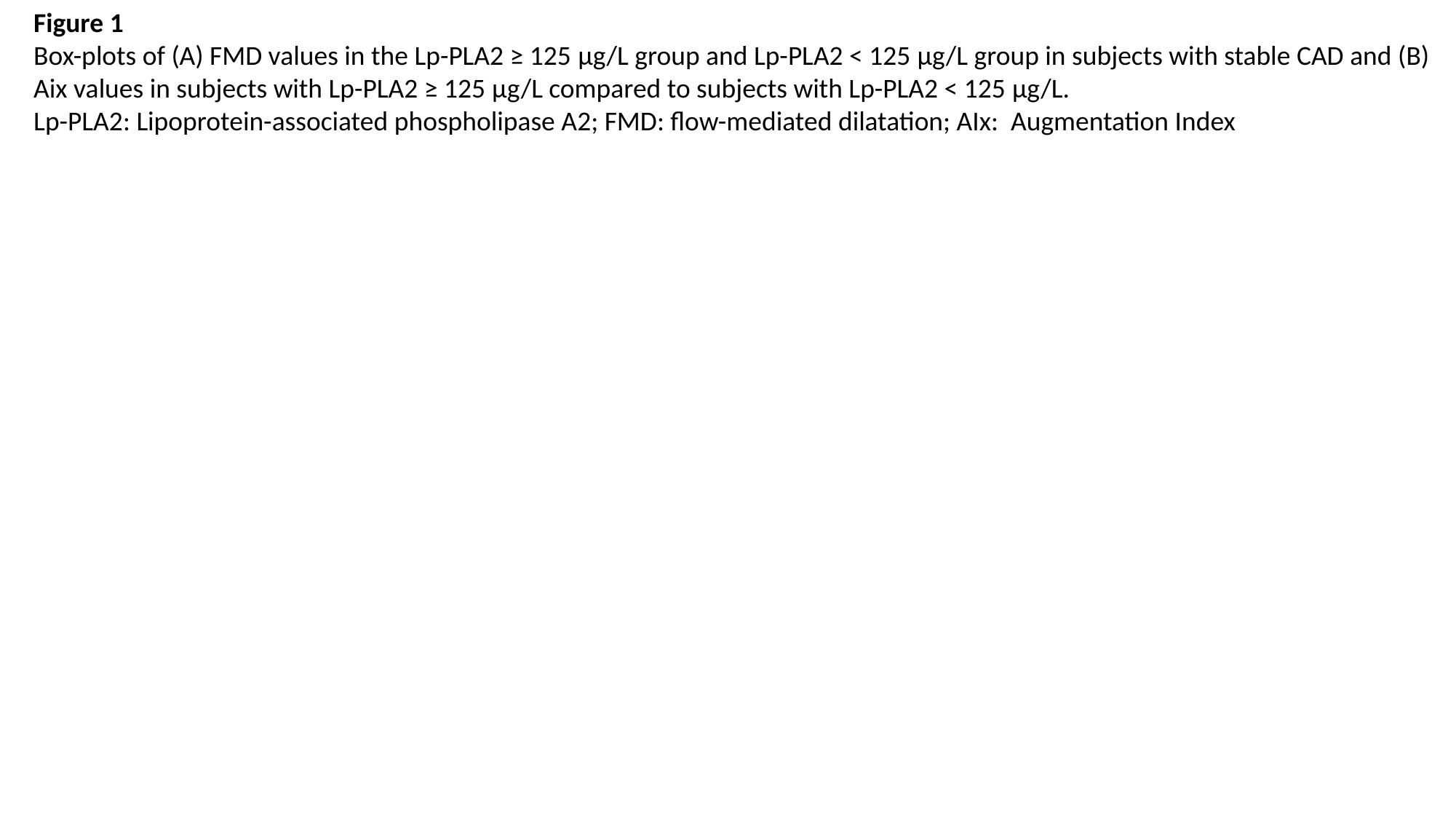

Figure 1
Box-plots of (A) FMD values in the Lp-PLA2 ≥ 125 μg/L group and Lp-PLA2 < 125 μg/L group in subjects with stable CAD and (B) Aix values in subjects with Lp-PLA2 ≥ 125 μg/L compared to subjects with Lp-PLA2 < 125 μg/L.
Lp-PLA2: Lipoprotein-associated phospholipase A2; FMD: flow-mediated dilatation; AIx: Augmentation Index
